# Tobacco Control as an LGBTQ+ Issue: Knowledge, Attitudes, and Recommendations from LGBTQ+ Community Leaders

**DOI:** 10.3390/ijerph18115546

**Published:** 2021-05-22

**Authors:** Veronica Acosta-Deprez, Judy Jou, Marisa London, Mike Ai, Carolyn Chu, Nhi Cermak, Shannon Kozlovich

**Affiliations:** 1 Department of Health Science, California State University, Long Beach, CA 90802, USA; veronica.acosta-deprez@csulb.edu (V.A.-D.); judy.jou@csulb.edu (J.J.); nhi.cermak@gmail.com (N.C.); 2 OUT Against Big Tobacco Program, Equality California, Los Angeles, CA 90010, USA; marisa.london@eqca.org (M.L.); mike@eqca.org (M.A.); 3 Urban and Regional Planning Masters Program, University of California, Los Angeles, CA 90095, USA; ctchu4@gmail.com; 4 Programs Department, Equality California, Los Angeles, CA 90010, USA

**Keywords:** tobacco, LGBTQ+, policy

## Abstract

Tobacco companies use price discounts, including coupons and rebates, to market their products. Lesbian, gay, bisexual, transgender, and queer (LGBTQ+) communities are targeted by these marketing strategies, contributing to inequitably high tobacco use. Some localities have adopted policies restricting tobacco price discounts; for successful implementation, community buy-in is crucial. From July–October 2018, Equality California staff conducted semi-structured interviews with seven participants in Los Angeles, CA. Themes included familiarity with tobacco price discounts, their perceived impact on tobacco use in LGBTQ+ communities, and attitudes toward potential policy restrictions. Interview notes were analyzed using a deductive approach to qualitative analysis. Awareness of tobacco price discounts varied; some interviewees were familiar, while others expressed surprise at their ubiquity. Price discounts were seen to disproportionately impact LGBTQ+ individuals, especially those who additionally identify with other vulnerable groups, including young people and communities of color. Support for policy restrictions was unanimous; however, interviewees expressed concern over political opposition and emphasized a need for culturally competent outreach to LGBTQ+ communities. Community organizations are essential in mobilizing support for policy reform. Understanding the perceptions and recommendations of community leaders provides tools for policy action, likely improving outcomes to reduce LGBTQ+ tobacco use through restricting tobacco price discounts.

## 1. Introduction

Cigarette smoking is the leading cause of preventable disease, disability, and death in the United States (US) [1]. Smoking prevalence has declined from 42% of US adults in 1965 to 14% in 2019 [2,3], mainly due to the decrease in smoking prevalence within higher income communities, while smoking remains prevalent in low income communities [4]. The number of deaths attributable to cigarette smoking still totals over 480,000 each year—equivalent to one in five deaths in the US [2,3]. Tobacco use is responsible for 90% of all lung cancer deaths and 80% of deaths from chronic obstructive pulmonary disease, as well as increasing the risk of coronary heart disease; stroke; and many cancer types, including oral and digestive tract cancers [5,6]. Economic losses associated with tobacco use are estimated at over $300 billion per year in direct medical costs and lost productivity [3,7].

Significant socio-demographic disparities in cigarette smoking and, more broadly, the use of tobacco products, persist across the US. Compared to the overall smoking prevalence of 14% among US adults, smoking is substantially more prevalent among adults who are aged 25–64 (17%); identify as non-Hispanic American Indian/Alaskan Native (21%) or non-Hispanic Other (20%); hold a general educational development (GED) certificate (35%), no high school diploma or equivalent (22%), or a high school diploma (20%); have annual household incomes of less than $35,000 (21%); or identify as lesbian, gay, bisexual (19–41%), or transgender (29–80%) [1,8]. In addition to use of traditional cigarettes, young adults are also much more likely to report using electronic cigarettes (e-cigarettes) (9% prevalence, compared to 5% among US adults overall) [1]. Despite sustained efforts aimed at supporting smoking prevention and cessation, young adults and youths under the age of 18 remain susceptible. An estimated 2000 young people below the age of 18 smoke their first cigarette each day, and 300 will go on to adopt a daily smoking habit [9].

A major driver of cigarette smoking, both traditional and electronic, is the pervasive marketing used by tobacco companies to promote their products. “Big Tobacco” spent an estimated $9.06 billion on the advertising and promotion of tobacco products in 2018, equivalent to around $25 million each day, or a little over $1 million every hour [7]. One favored tactic is the use of price discounts, including coupons, gift certificates, rebates, and other measures aimed at lowering the price of tobacco products to consumers; together, these types of price discounts comprise nearly 75% of all cigarette marketing. Aggressive marketing strategies, including price discounts, are often targeted toward vulnerable populations. In particular, lesbian, gay, bisexual, transgender, and queer (LGBTQ+) communities were identified as a “new market with growth potential” starting in the 1990s [10]. LGBTQ+ individuals are at higher risk of smoking due to the stresses associated with their sexual orientation and/or gender identity, including social stigma and discrimination; fear of rejection by family and peers; lack of access to culturally competent health care services; and, in some cases, internalized homophobia [11,12,13,14]. Deliberate efforts by the tobacco industry to market their products to the LGBTQ+ community compound the effects of these risk factors. Since the 1990s, tobacco companies have targeted gay and lesbian publications with their advertisements, often by depicting smoking as a normalized part of the LGBTQ+ lifestyle [15,16]. One 2008 study found that 30% of non-tobacco advertisements in LGBTQ+ publications featured tobacco use [17]. Tobacco products are also heavily promoted at LGBTQ+ venues and events, including LGBTQ+ pride festivals, bars and clubs, and community centers and organizations [18]. These marketing strategies have had a large impact on younger LGBTQ+ adults in particular; compared to 12% of heterosexual adults ages 18–34, 19% of gay/lesbian, 17% of bisexual, and 33% of transgender young adults self-identify as current smokers [19].

Recognizing the harmful impact of tobacco, policymakers have enacted several reforms aimed at regulating tobacco sales, marketing, and use. Following the 1964 publication of the first report on smoking by the US Surgeon General, governing bodies at local, state, and national levels have adopted such policies as health warnings on cigarette packaging; designated “smoke-free” zones, particularly around schools and public buildings; prohibitions on the use of characters to advertise tobacco products to children; heavy excise taxes to raise the price of cigarettes for consumers; and funding for tobacco control and smoking cessation programs [20,21]. While these efforts have been successful in some aspects, including an overall decrease in smoking prevalence in the general population, several points of concern remain. Tobacco companies regularly lobby against regulation and, failing to block policies such as excise taxation and minimum cigarette pricing, circumvent legislative restrictions by adopting aggressive price discounts [22,23]. The rising popularity of e-cigarettes presents another challenge, as they are exempt in many cases from regulations targeting traditional forms of tobacco use.

While some localities have adopted restrictions on some aspects of price discounting, such as prohibiting the distribution of free samples, legislation remains patchwork. The successful adoption and implementation of such policies often depend on support from local officials such as city council members and health departments, as well as grassroots support from those most affected by these types of marketing strategies, including the LGBTQ+ community [22,24]. To date, there has been little research examining the process of gaining local-level support among LGBTQ+ communities for policies restricting or ending the use of price discounts on tobacco products. Though outreach to LGBTQ+ individuals may follow many of the same community-based strategies that have been found effective more generally [25,26], it is important to understand the unique experiences and perspectives of this community, including its history of being targeted by tobacco companies for aggressive marketing, in order to identify effective strategies for policy advocacy.

The aim of this paper is to examine in greater depth the knowledge and attitudes of community leaders from various community agencies in Los Angeles County, California (CA), around tobacco use and marketing within LGBTQ+ communities. Community-based organizations play a valuable role in promoting the health of their communities through a variety of activities. However, research on the process of how these organizations have been instrumental in effecting change in their communities, particularly in their engagement in tobacco control activities, has been severely lacking. Since tobacco is the number one cause of preventable death in the United States, and its impact on hard-to-reach and diverse communities has been severe, the perceptions of leaders and key personnel in community agencies that serve minority communities are an important starting point for receptivity to future tobacco control activity by the organizations.

This study also examines interview participants’ attitudes and beliefs about the government’s role and interest in working as advocates on tobacco control policies, as well as some of the issues around the barriers and advantages of a potential citywide flavored tobacco policy. The methodology described here is a “rapid appraisal” technique of participant interviews for assessing community leaders’ perceptions. In doing so, this study aims to provide much-needed information on the specific needs of LGBTQ+ communities, as well as enhanced understanding and support for advocacy efforts against tobacco marketing. While it has been established that the high rates of tobacco use within the LGBTQ+ community are due, in part, to the aggressive marketing by tobacco companies that sponsor events, bar promotions, giveaways, and advertisements [27,28,29,30,31], there is a general lack of knowledge about the perspectives of the LGBTQ+ community, particularly those recognized as community leaders, when it comes to the actions of Big Tobacco. The interviews conducted for this study aim to provide much needed insight into the opportunities and challenges for engaging LGBTQ+ community leaders in tobacco-related health equity policy work.

## 2. Materials and Methods

The qualitative research method was used to collect data in this study. This method was chosen because the approach provides an opportunity to comprehensively explore the experience of participants, which is known as the phenomenological approach (e.g., humanistic approach). The aim of qualitative research is to understand the social reality of individuals, groups, and cultures as it is perceived by participants who feel or live it. Qualitative research is recognized for its ability to add a new dimension to interventional studies that cannot be obtained through the measurement of variables alone [32,33]. In using the qualitative method (vs. the quantitative method), the aim was to learn “how” and “why” a particular phenomenon, behavior, attitude, or perception operates in a particular context. This method was used to generate hypotheses and theory from the data. 

This study was developed to recruit individuals who fit narrow and specific characteristics related to the participants’ professional lives [33], inclusive of community leaders who serve or work with lesbian, gay, bisexual, transgender, and queer (LGBTQ+) people, including those who work for LGBTQ+ or allied organizations in some way. This included outreach to public sector employees (excluding elected officials and their office staff), leaders of LGBTQ+ serving organizations, and leaders of allied organizations; allied organizations are defined as organizations that recognize that they serve target communities that are inclusive of LGBTQ+ people. The snowball sampling for a hard-to-reach population recruitment strategy was employed as defined by Goodman [34], where an initial set of 4 participants helped to identify the additional 14 potential participants.

From July to October 2018, participants influential in LGBTQ+ communities in Los Angeles County, CA, were identified and asked to participate in a brief interview about their perceptions around tobacco marketing and policy; none of the remaining 11 possible participants responded to our follow-up invitation two weeks later. Interviews were conducted by members of our study team either via telephone or via an online service such as Skype or MSN Messenger. To examine the interviewees’ perceptions on tobacco marketing and policy, especially pertaining to LGBTQ+ communities, a semi-structured interview guide was developed by the study team. Each interview consisted of 12 open ended questions covering 5 topic areas and lasted around 15–45 min. The questions covered the following: (1) knowledge, attitudes, and behaviors about the tobacco industry’s use of handouts such as samples, coupons, gift cards, and other price reductions; (2) perceptions and opinions about policy that would restrict or end the use of tobacco price reductions; (3) perceived challenges or barriers to policy adoption; (4) reasons for support or opposition to policy interventions; and (5) strategies for future policy interventions. The interview questions covered familiarity with the topic(s), perceptions of the topic(s), perceived impact of the topic(s), and personal experiences with the topic(s). Key demographic characteristics, including gender and years in their current position, were also collected at the time of interview. Participants were informed of the purpose and contents of the interview at the beginning, and were encouraged to pause, stop, or withdraw from the interview if they felt uncomfortable with any question. Handwritten notes were taken during each interview, with names and identifying information removed prior to analysis in order to maintain confidentiality.

After all seven interviews were completed, the notes taken by the interviewer during each interview were analyzed using a deductive thematic analysis approach to qualitative data analysis. This involved reviewing the interview notes for 5 key themes generated from the instrument topics: knowledge, perceptions, impact, policy views, and recommendations. Each theme was examined to gain an understanding of the participants’ perceptions of and experiences with tobacco marketing and policy. Any disagreements in interpretation were discussed between team members and resolved using an iterative approach.

## 3. Findings

Key informant interviews were completed with a total of seven participants across three cities in Los Angeles County. Interviewees represented a variety of community and public service organizations. Out of the seven interviewees, five self-identified as male, and two self-identified as female. On average, interviewees had been serving in their current positions for just over three years. Table 1 shows the key demographics of the interviewees.

### 3.1. Knowledge and Perceptions of Tobacco Marketing Using Price Discounts

Participants’ familiarity with marketing strategies involving the use of coupons, rebates, and other price discounts by tobacco companies was wide-ranging. Of the seven interviewees, two stated that they were “Not familiar” with the issue; two stated that they were “Somewhat familiar”; and three stated that they were “Very familiar”. Those who were very familiar described how tobacco companies have been “really generous with coupons” since the tax increase, and they, as well as their friends, have taken advantage of the market by using coupons to buy tobacco products.

When asked their opinion on the use of aggressive marketing strategies by tobacco companies, including coupons, rebates, and other price discounts, most participants expressed surprise or concern at the pervasiveness of such tactics. One participant recounted their (To maintain the confidentiality of interviewees, all gendered pronouns have been replaced by gender-neutral “their”, “they”, or “them”) surprise to learn that a friend was saving cigarette packaging tops and mailing them in to receive rebates on their purchases, stating:

*“When I first found out a friend was saving cigarette packaging tops and mailing it in, I was shocked. They were quite a savvy individual, and I felt they fell prey to this marketing tactic. They wanted to get a Marlboro tent. They live in the city and don’t even camp. It surprised me that they were hooked by this marketing tactic.”*

Another participant stated that they knew many people who use tobacco products, but had been surprised when they received coupon “foldouts”. The participant went on to add that, although they understood the negative health effects of tobacco use, they did not want to risk their friendships by opposing their friends’ smoking habits.

Some of the interviewees discussed tobacco marketing in the context of their professional roles. One participant described their work with older adults at a home health agency. This interviewee explained that several seniors in the home who used tobacco also had histories of strokes or cancer, which the interviewee noted could have been attributed to tobacco use.

Two participants expressed strong opposition to the marketing strategies used by tobacco companies. Both interviewees explained that they smoked in their younger years but now find tobacco companies aggressive and “violent” in their marketing tactics. While both interviewees expressed appreciation for the decreasing prevalence of smoking in general, they noted that many people still smoke. As one interviewee remarked, “I think in my time, a decrease in smoking has occurred, but with the advent of vaping, it’s increasing again. It’s giving opportunity for more people to smoke again”.

### 3.2. Populations Perceived to Be Most Affected by Tobacco Marketing Strategies

Most participants expressed concern about the negative effects of tobacco use on diverse groups, especially young people, older adults, socio-economically disadvantaged populations, and people of color. When participants were asked who they perceived to be most affected by aggressive tobacco marketing strategies, their responses ranged widely and included the LGBTQ+ community, seniors, people with fixed or lower incomes, undocumented people, young people, and communities of color.

In particular, young people were described as a vulnerable population due to being characterized as nonchalant about the negative effects of tobacco; spending their resources lavishly; conscious of their social standing; easily prone to peer pressure; and, most of all, seen as easy targets on the path toward addiction. One participant noted that young people were more prone to tobacco use “to be social, to be seen as cool”. Another, who worked with at-risk youth, expressed deep concern for their well-being, noting the susceptibility of this population to marketing due the emotional volatility associated with the teenage years. The interviewee explained that many of the young people they worked with had already experienced alcohol and drug addiction, and tobacco presented another potential source of addiction.

Additionally, a majority of interviewees (71%) cited communities of color as a target of tobacco marketing. Five of the seven also mentioned people who were in lower- or lower-middle socio-economic strata as a targeted population.

### 3.3. Perceived Impact of Tobacco Marketing on LGBTQ+ Populations

When asked whether tobacco marketing strategies had a significant impact on LGBTQ+ communities, five of the seven interviewees (71%) said yes. Of the five, three strongly believed LGBTQ+ populations to be a clear target. One participant described a popular tobacco company cartoon camel mascot as an example of how marketing strategies focused on the LGBTQ+ population, citing an appeal to the leather community with the clothing choice and the phallic face structure. Interviewees conveyed that a variety of factors contributed to LGBTQ+ communities’ vulnerability to tobacco marketing, including the daily stress of coping with prejudice and stigma. One participant attributed smoking among LGBTQ+ youth to acceptance associated with a culture of partying. For LGBTQ+ adults, opportunities to smoke often presented themselves at bars and clubs. Even the two participants who answered no expressed awareness of increased rates of tobacco use in LGBTQ+ communities and mentioned that advertisements for tobacco products in LGBTQ+ publications are likely to be pervasive. As one interviewee stated, “I don’t know enough in this area, [but] we know that the LGBT community in West Hollywood has a higher percentage of smokers”.

Interviewees were asked about their personal encounters with tobacco marketing strategies such as coupons, rebates, and other price discounts. Of the seven interviewees, four (57%) had been approached with some sort of price-related tobacco marketing or knew someone who had, while three interviewees had not. Several participants had witnessed the distribution of materials such as coupons and rebates during community events at a park or beach, in movie theaters, at LGBTQ+ Pride events, and in LGBTQ+ publications and magazines. One participant recalled that they were offered coupons for tobacco products at a Pride event in the previous year. Another recounted “free vaping” being available at an “outdoor concert festival” they had attended:

*“They were giving [vaping cartridges] away at this tent. […] They were giving it away and people were just going crazy. I don’t think they were asking questions, people were just adopting immediately.”*

Given their personal experiences, four out of the seven interviewees believed that tactics such as offering coupons, rebates, and other price discounts were effective in promoting tobacco use in their communities. As one participant noted, “we don’t receive a lot of free stuff just for being LGBT, and we’re not wise to some of these tactics,” making the tactics more effective.

A majority of the interviewees discussed issues around intersectionality. Six (86%) of the seven participants expressed concern about tobacco marketing targeted toward individuals in lower socio-economic strata and youth, and especially toward LGBTQ+ individuals who also fell into those categories. Several interviewees noted that LGBTQ+ individuals comprised a part of every community, and if they belonged to another high-risk group, such as those with lower income, women, people of color, or individuals with mental health conditions, they may be doubly burdened and therefore more susceptible to addiction and other tobacco-related diseases. Several participants compared the marketing strategies used by tobacco companies to those used by producers of unhealthy foods and beverages, which have disproportionately affected vulnerable populations. Interestingly, one participant noted that wealthy LGBTQ+ individuals were less likely to be affected by price discounts, as “they don’t care if they’ll pay $10 a pack”.

### 3.4. Views on Policy Restrictions or Prohibition of Tobacco Marketing Using Price Discounts

When participants were asked their views on a citywide policy that prohibited or restricted the use of tobacco marketing strategies involving coupons, rebates, and other price discounts, all seven (100%) respondents expressed support.

Several interviewees discussed the potential advantages of such a policy. One major advantage was that prohibiting or restricting price-based marketing would reduce tobacco use in the general public by raising the cost of tobacco products. A related advantage was the health benefit that would likely result from decreased tobacco use, especially among vulnerable populations such as individuals with lower incomes, LGBTQ+ communities, or young people, who tend to be targeted most actively by tobacco marketing. One interviewee noted that a policy of this sort “certainly reduces industry’s ability to hook a younger market, if it included education for youth and adults that this has health implications”. Participants also expressed support for a policy that would reduce the early adoption of a smoking habit, particularly among LGBTQ+ youth, who have become one of the communities most affected by tobacco-related diseases. One interviewee noted that restrictions on marketing may decrease the appeal and acceptability of tobacco use to LGBTQ+ consumers.

Interviewees were asked to identify potential disadvantages and/or challenges to adopting said policy. Most respondents noted that attempts to regulate behavior through policy would infringe on individual freedoms and the right to choose, which may be particularly resonant with LGBTQ+ communities due to an emphasis on advocating for the right to bodily autonomy. As one interviewee observed, “I can see members in the community saying it’s a free market, we’re adults, [tobacco products] aren’t available to kids and people should be able to make up their own mind. Limiting consumer freedom and such”.

Interestingly, one participant perceived that government regulation focusing on behavior may limit children’s perceptions around choice, which may lead to weakened decision-making skills in the future. Another highlighted the difficulty of earning support from marginalized subgroups within communities. Several participants voiced questions about what such a policy would entail; for example, most interviewees wanted to know the exact practices and/or products that would be regulated. Many also voiced concern about the economic impact of such restrictions, especially on small businesses. A related economic challenge identified by some participants was the lack of funding and resources for successfully implementing such a policy, including identifying and enforcing penalties against infractions, as well as educating the public to ensure awareness of and compliance with the policy.

Participants also spoke to the difficulty of garnering political support for this type of policy. One interviewee specifically mentioned the tenuous politics around the interests of government, businesses, and the voting public, noting:

*“The tobacco industry and the amount of money they have [would be a barrier]. I see a challenge would also be getting locally elected politicians. It would take a lot of education and advocacy to move that political will. But the challenge will be in the opposition, they are well funded and they have much more of ability to manipulate public opinion”*

Another commented on the difficulty of gaining support from city council members and educating them about the negative impact of tobacco marketing tactics on their constituents’ health. The complexity of the policymaking process was also mentioned as a potential barrier, with one interviewee noting that getting any type of policy signed, or even deliberated, “moves slower than cold molasses”.

Potential opposition from the tobacco industry was also cited as a concern. One participant described “lobbying from Big Tobacco” as a potential challenge, elaborating:

*“LA is one of the largest cities in the country. We’d see big money coming in to do massive billboard campaigns, investing in it so LA doesn’t become that first […] national example. I would imagine if I was one of the big tobacco companies, I wouldn’t want to see restrictions on this. It would have an impact of millions of dollars for them.”*

### 3.5. Recommendations for Policy and Outreach

Despite these challenges, many of the interviewees were personally willing to advocate for a policy that restricted or prohibited the use of price discounts as a tobacco marketing strategy, as the benefits were perceived to outweigh the disadvantages. One participant recommended placing “as many obstacles as possible…to make it as hard as possible for people to get to tobacco,” including geographic restrictions on coupon redemption. Overall, participants voiced interest in expediting regulation before youth and individuals from targeted communities begin to smoke, so as to prevent the long-term impact of tobacco use. One interviewee expressed willingness to share information via social media and speak with the Los Angeles City Health and Human Services Commission regarding related issues.

Interviewees offered several recommendations for gaining policy support. One suggested strategy involved outreach to city council members and staff; in some locations, such as West Hollywood, outreach to organized entities such as the Department of Health and the Chamber of Commerce was also recommended due to their strong influence on city politics. One participant suggested using statistics to demonstrate that a proposed policy would promote health without harming businesses in the community. Overall, community involvement was a strong theme among most participants. Interviewees suggested outreach to civic leaders in order to promote community engagement and buy-in. Suggested ways to boost community engagement included media outreach that focuses on changing social norms; working directly with youth and parent groups; and targeted education campaigns for vulnerable populations such as the Asian-American and Latinx communities, LGBTQ+ community, and individuals with low socio-economic status. One interviewee recommended educational strategies with broader reach, such as fotonovelas for Latinx communities and soap operas for Asian-American communities, noting that “telling a story is always helpful”. Another recommendation for improving community buy-in was to introduce policies incrementally by, for instance, limiting restrictions on price discounts to a smaller geographic area before proposing any city- or county-wide policies.

Specific recommendations for outreach to LGBTQ+ communities focused on education and trust-building. Interviewees emphasized the need to educate communities about the “true impact” of tobacco use on the LGBTQ+ population by addressing reasons why LGBTQ+ individuals are more likely to smoke, correcting any misinformation about price discounting strategies, and highlighting resources available to help with tobacco cessation. Participants noted that positive messaging may be more effective with this population, such as emphasizing the health benefits of tobacco cessation and providing more resources to support individuals who want to quit. As one interviewee noted:

*“Scare tactics don’t work. Positive reinforcement is good. Showing positive images of people enjoying their lives. Focus on cost savings by not smoking or by stopping smoking. Focus on harm reduction.”*

Several interviewees noted the importance of building trust by elevating voices within LGBTQ+ communities from those who have been affected by “Big Tobacco”. Participants also emphasized a tailored approach to both education and advocacy that is culturally sensitive and appropriate, given the unique experiences and needs of the LGBTQ+ community. One interviewee noted that messaging “should be from a positive perspective” and framed to address the question of, “As an LGBTQ person, what does this mean to not smoke, for the health of my body?”

## 4. Discussion

Though price discounts comprise a large majority of tobacco industry efforts to market their products [7,35,36], the results of our study suggest that awareness about the use and impact of price discounts such as coupons, rebates, and gift certificates is not uniform, even among community leaders and advocates in a large metropolitan area such as Los Angeles County. While some of our study participants were familiar with such strategies, several were not, and some were surprised at their pervasiveness. However, even participants who were less familiar with price discounts were generally aware that tobacco marketing strategies focus heavily on LGBTQ+ communities, and several reported personal encounters with free or discounted products at events or venues specific to LGBTQ+ interests. This aligns with prior research showing that up to half of current adult smokers in the US have used some type of price discount when purchasing tobacco products [37,38,39,40], and that younger adults and LGBTQ+ individuals tend to be more prolifically targeted by these marketing strategies [41,42,43]. In addition, our findings highlight the need for intersectional approaches to understanding the distribution and impact of tobacco price discounts. Several participants emphasized the overlap between the LGBTQ+ community and other vulnerable populations, including youth, individuals with lower incomes, and communities of color. Intersectionality in research, community education, and policy interventions is needed to address the unique risk factors experienced by LGBTQ+ people.

This study finds that LGBTQ+ communities face unique risk factors that may increase their receptiveness to price discounting strategies such as coupons and rebates. Recent research has found that LBGTQ+ people are more likely to not only have exposure to tobacco marketing and price discounts, but also to actively engage with these offers by accepting and using them to purchase discounted tobacco products, searching for them online, or sharing them via social media [43,44]. Similar to prior studies, ours found that LGBTQ+ individuals may be more susceptible to price discounting strategies due to tobacco companies’ targeting of LGBTQ+-specific events and venues [11], which has the effect of normalizing tobacco use within these communities. An additional contributing factor we identified is the frequent exclusion of LGBTQ+ individuals from non-tobacco product promotions of all types, making tobacco promotions aimed at them more attractive. Moreover, social norms against the use of tobacco coupons or rebates may be lacking, as several interviewees expressed either surprise at their friends’ use of such offers or hesitance to oppose it. Additional interventions that support awareness and communication around the use of price discounts may be needed to address these risk factors.

We found unanimous support among our interviewees for local policies that limit the use of price discounts, and, similar to examples from other jurisdictions [45,46], interviewees emphasized the need for engagement with broad coalitions of community members and organizations. Again, intersectionality was emphasized; while previous successful campaigns for policy change have targeted outreach primarily to, for instance, LGBTQ+ communities and venues [45], our results suggest that the diversity of localities such as the Los Angeles County area necessitates multi-faceted and intersectional outreach initiatives that speak to individuals who belong to both LGBTQ+ and other vulnerable communities. Additionally, messaging that focuses on the health benefits of reduced tobacco use as a result of such policies is viewed as being most effective in garnering support, especially if it highlights prevention among LGBTQ+ youth.

Our findings around the challenges associated with policy reform also align with prior research. Participants raised questions around the specific provisions of any proposed policy, including whether products would be prohibited versus restricted, as well as the specific products and practices included, reflecting similar conversations that have occurred at the state level in California [47]. Concerns about the impact of policy restrictions on local businesses, as well as pushback from industry, are also similar to policy efforts around restrictions on tobacco products and other products harmful to health such as sugar-sweetened beverages [48,49]. Additionally, both political and administrative feasibility were identified as potential barriers by our study participants [50]; specifically, there was consensus around the need to actively engage city council members, as well as secure resources for communication and enforcement around any adopted policies. We also identified unique challenges in gaining grassroots support, as LGBTQ+ communities have been shown to be resistant to policy-based interventions [8], thought to be due to their history of advocacy for bodily autonomy. A strong emphasis on cultural competency, inclusiveness, and horizontal messaging within communities were all recommended in order to address the complex needs of LBGTQ+ communities.

The results of this study should be considered in light of several limitations. First, the relatively small sample size of seven interviewees means that their views may not be widely representative of community leaders in the LGBTQ+ and/or tobacco prevention spaces. Traditionally hard-to-reach populations of study participants often have a lower rate of responding to participation requests. Due to the use of the snowball sampling technique, many of the 11 potential participants who did not respond to recruitment emails were referred by other participants, and may not have had an established trust relationship with the study. Moreover, these results specifically address the conditions and needs of communities in the Los Angeles County area and may not be generalizable to other locations, especially those with a different demographic and political makeup. Similarly, the interview participants are likely not representative of all community leaders in Los Angeles County. Future research should aim to engage with a larger number of community stakeholders from a wider geographic area. These interviews did not include policymakers such as city council members or their staff. While this was done to keep the focus of the interviews on community leaders, it is also important to examine the knowledge, attitudes, and preferences of decision-makers around this topic, and we recommend additional research that does so. Lastly, these interviews did not include the general public who were not leaders/activists. As seen from Los Angeles County community survey data [8], the general public’s views differ from those of community leaders. These differences add to the emphasis on community education campaigns being necessary for policy initiatives. Despite these limitations, these interviews provide unique insight into the knowledge, attitudes, and potential for action around tobacco marketing using price discounts in a traditionally underserved community. By doing so, we identify specific recommendations for engaging LGBTQ+ people and the diverse communities to which they belong, as well as the policymakers and organizations who serve them, in support of policy action that may ultimately reduce the prevalence of tobacco use in LGBTQ+ youth and adults.

## 5. Conclusions

LGBTQ+ youth and adults experience a higher prevalence of tobacco use, in part due to targeted efforts by the tobacco industry to market their products to these communities. One of the most pervasive marketing tactics for tobacco products is the use of price discounts such as coupons and rebates. Community leaders active in LGBTQ+ communities and anti-tobacco advocacy from Los Angeles County identified unique risk factors that make LGBTQ+ communities more susceptible to price discounting strategies, as well as providing insight into the opportunities and challenges around policy interventions that would restrict or prohibit price discounting tactics. Culturally competent outreach to LGBTQ+ communities, with consideration for intersectionality, is needed in order to successfully engage community members in advocating for policy change to reduce the rates of tobacco use and promote long-term health equity.

## Figures and Tables

**Table 1:** Key characteristics of the interview participants.

Interview Number	Type of Organization	Length of Time in Current Position
1	LGBTQ+ Organization	6 years
2	Public Sector	3 years
3	Public Sector	1 year
4	Public Sector	3 years
5	Public Sector	2 years
6	Community Organization	5 years
7	Public Sector	Declined to state

## Data Availability

Not applicable.
